# Gas Chromatography-Mass Spectrometry Analysis of *Ulva fasciata* (Green Seaweed) Extract and Evaluation of Its Cytoprotective and Antigenotoxic Effects

**DOI:** 10.1155/2015/520598

**Published:** 2015-11-03

**Authors:** Idania Rodeiro, Sitlali Olguín, Rebeca Santes, José A. Herrera, Carlos L. Pérez, Raisa Mangas, Yasnay Hernández, Gisselle Fernández, Ivones Hernández, Sandra Hernández-Ojeda, Rafael Camacho-Carranza, Ana Valencia-Olvera, Jesús Javier Espinosa-Aguirre

**Affiliations:** ^1^Departamento de Farmacología, Centro de Bioproductos Marinos (CEBIMAR), Loma y 37, Vedado, Plaza de la Revolución, 11300 La Habana, Cuba; ^2^Departamento de Medicina Genómica y Toxicología Ambiental, Instituto de Investigaciones Biomédicas, Universidad Nacional Autónoma de México (UNAM), CP 70228, 04510 México, DF, Mexico; ^3^Universidad de la Habana, Zapata y G, Vedado, Plaza de la Revolución, 10400 La Habana, Cuba; ^4^Departmento de Bioquímica, Instituto de Ciencias Básicas y Preclínicas (ICBP) “Victoria de Girón”, Avenida 146, Playa, 11300 La Habana, Cuba

## Abstract

The chemical composition and biological properties of *Ulva fasciata* aqueous-ethanolic extract were examined. Five components were identified in one fraction prepared from the extract by gas chromatography-mass spectrometry, and palmitic acid and its ethyl ester accounted for 76% of the total identified components. Furthermore, we assessed the extract's antioxidant properties by using the DPPH, ABTS, and lipid peroxidation assays and found that the extract had a moderate scavenging effect. In an experiment involving preexposition and coexposition of the extract (1–500 *µ*g/mL) and benzo[a]pyrene (BP), the extract was found to be nontoxic to C9 cells in culture and to inhibit the cytotoxicity induced by BP. As BP is biotransformed by CYP1A and CYP2B subfamilies, we explored the possible interaction of the extract with these enzymes. The extract (25–50 *µ*g/mL) inhibited CYP1A1 activity in rat liver microsomes. Analysis of the inhibition kinetics revealed a mixed-type inhibitory effect on CYP1A1 supersome. The effects of the extract on BP-induced DNA damage and hepatic CYP activity in mice were also investigated. Micronuclei induction by BP and liver CYP1A1/2 activities significantly decreased in animals treated with the extract. The results suggest that *Ulva fasciata* aqueous-ethanolic extract inhibits BP bioactivation and it may be a potential chemopreventive agent.

## 1. Introduction 

Marine seaweeds have been harvested for several years in the Far East and Asia Pacific countries, where they are consumed as food. In the last decade, this practice has also extended to North America and Europe [1]. At present, the economic potential of the seaweed industry is widely recognized [2]. In addition, seaweeds are considered an attractive avenue for the screening of biologically active compounds, due to their biodiversity and safety [2, 3].

Until now, the seaweed species studied have shown variations in their chemical composition (proteins, carbohydrates, lipids, minerals, and vitamins) associated with the influence of environmental factors such as seasonal periods, temperature, light, salinity, location, and storage conditions [4]. Seaweeds are able to produce secondary metabolites with interesting bioactive properties, including antibacterial, antifungal, antiviral, and antioxidant effects [5–9].* Ulva fasciata *Delile, also known as sea lettuce, grows abundantly along coastal seashores. The antioxidant and antibacterial properties of this seaweed have been previously reported [10, 11].

Many extracts or partially purified polysaccharides from various algae have shown antitumor activity against experimental tumors in animal models [2, 12, 13]. The mechanism underlying this effect could be related to their antioxidant properties or control of cell proliferation. Additionally, Ryu et al. [14] showed that the ethanolic extract of* U. fasciata* has anticancer activity associated with the modulation of apoptotic signals, including mitochondria- and caspase-dependent processes, in human colon cancer HCT116 cells.

Cancer is a serious global health problem and the primary cause of morbidity and mortality in Cuba [15]. Thus, the search for novel nutraceuticals with potential benefits for the prevention or therapy of cancer is well justified. An aqueous-ethanolic extract of* U. fasciata *collected from the north coast of Cuba was obtained and analyzed. Phytochemical study of this extract shows a high content of chlorophyll b, carotenoids, protein, carbohydrates, fiber, Ca, Mg, K, Fe, Zn, Cr, and Mn [16].

In order to contribute to the chemical characterization of the* U. fasciata* extract, here we report some nonpolar constituents of the extract. A chloroform-diluted fraction of the extract was prepared and its composition was determined by using gas chromatography-mass spectrometry (GC-MS). We also studied the protective effects of the whole extract by assessing its ability to protect against benzo[a]pyrene- (BP-) induced cytotoxicity in C9 hepatic cells in mice. The antioxidant capacity and inhibitory effects of* U. fasciata* on CYP1A1/2 and CYP2B1/2 activities involved in the metabolism of several human mutagens/carcinogens were also investigated.

## 2. Materials and Methods

### 2.1. Chemicals

Analytical-grade reagents and reference substances were obtained from Aldrich (Milwaukee, MN, USA). Phenobarbital (PB) was purchased from Abbott Laboratories (Mexico City, Mexico). Beta-naphthoflavone (*β*-NF), resorufin, 7-ethoxyresorufin (ER), methoxyresorufin (MR), benzyloxiresorufin (BR), pentoxyresorufin (BR), NADPH, BP, 2,2-diphenyl-2-picrylhydrazyl (DPPH), 2,2′-Azino-bis(3-ethylbenzothiazoline-6-sulfonic acid) (ABTS^•+^), thiobarbituric acid (TBA), corn oil, and Giemsa stain were purchased from Sigma Chemicals Co. (St. Louis, MO, USA). Microsomes of* Baculovirus* expression systems from rat CYP1A1-expressing insect cells (Supersomes) were purchased from BD-Gentest (Woburn, MA, USA).

### 2.2. Material

*Ulva fasciata* Delile (Chlorophyta) was collected from the estuary of Quibu River in Cuba (82°27′48′′W and 23°53′04′′N). The seaweeds were collected by hand from the intertidal zone in October 2013. After collection, the materials were immediately washed to remove epiphytes and sand and transported to the laboratory. After washing with distilled water, the samples were dried at 60 ± 1°C to constant weight, milled, and stored desiccated in plastic receptacles. Fifty grams of dried* U. fasciata* powder was continuously macerated with 500 mL of ethanol : H_2_O (1 : 1 vol/vol) for 24 h at room temperature. The extract obtained was filtered and concentrated to dryness under reduced pressure at 45°C.

### 2.3. Gas Chromatographic/Mass Spectrometric Analysis

One hundred milligrams of the dried extract was partitioned within CHCl_3_/H_2_O (1 : 1 v/v). The resulting crude organic phase was filtered and concentrated to dryness under reduced pressure at 45°C by using a rotary vacuum evaporator. Then, the fraction obtained was analyzed by gas chromatography-mass spectrometry (GC-MS).

The analyses were performed using a GC-MS system (Model QP 2010 series, Shimadzu, Tokyo, Japan) equipped with an autosampler model AOC-20i and an RTX-1 fused silica capillary column of 30 m in length, 0.25 mm in diameter, and 0.1 *μ*m of film thickness. The column oven temperature was programmed from 50 to 300°C for 2°C min^−1^. Ionization of the sample components was performed in electron impact mode (EI, 70 eV). The temperature of the injector was fixed at 300°C and that of the detector at 310°C. Helium (purity, 99.9995%) was the carrier gas; its flow rate was fixed at 1 mL min^−1^. The mass range from 40 to 1000* m/z* was scanned at a rate of 3.0 scans/s. One microliter of the organic extract of* U. fasciata* was manually injected into the GC-MS system by using a Hamilton syringe, for total ion chromatographic analysis by split injection (1 : 40). The total running time of the GC-MS system was 15 min. The relative percentage of each extract constituent was expressed as percentage with respect to peak area normalization. The conversion of analog data to digital data was performed using the GC Solution software.

### 2.4. Antioxidant Study

#### 2.4.1. Assay of 2,2-Diphenyl-2-picrylhydrazyl (DPPH^•^) Scavenging Activity

The antioxidant capacity of the extract was measured as DPPH radical scavenging ability according to the method described by Tabart [17] with minor modifications. DPPH (1500 *μ*L) in ethanol (0.075 mg/mL) was mixed with 750 *μ*L of the extract at five different concentrations (10–1500 *μ*g/mL). A control sample (ethanol) and a reference sample (ethanol plus DPPH) were used. The antioxidant ascorbic acid was used as a positive control. The decrease in the absorbance (Abs) at 515 nm was determined using a UV-1201 spectrophotometer (Shimadzu, Japan), until the reaction plateau step was reached. The IC_50_ values were determined, where they represent the concentration of extract that caused 50% inhibition of the maximum effects, and the scavenging effect was calculated as a percentage of DPPH scavenged, where % DPPH inhibition = (control Abs − sample Abs)/(control Abs) × 100, control Abs = ethanol + DPPH Abs and sample Abs = sample + DPPH Abs.

#### 2.4.2. Assay of 2,2′-Azino-bis(3-ethylbenzothiazoline-6-sulfonic Acid) (ABTS^•+^) Radical Scavenging Activity

The scavenging activity of* U. fasciata *extract was also tested as previously described [18]. Briefly, ABTS^•+^ solution (7 mM) was mixed with potassium persulfate (2.45 mM) and stored during 16 h in the dark to generate the ABTS^•+^ radical cations. Then, the ABTS^•+^ absorbance was adjusted to 0.70 ± 0.02 at 734 nm. Free radical scavenging activity of* U. fasciata* was assessed by mixing 300 *μ*L of test sample with 3.0 mL of ABTS^•+^ radical solution. The decrease in absorbance at 734 nm was measured after six minutes. The percentage inhibition was calculated as follows: Scavenging activity (%) = (control Abs − sample Abs)/(control Abs) × 100. The antioxidant capacity of* U. fasciata *was expressed as IC_50_ values (*μ*g/mL).

#### 2.4.3. Assessment of Lipid Peroxidation

The capacity of the inhibition of liver lipid peroxidation by* U. fasciata *extract was assessed as described elsewhere [19] with slight modifications. The livers of male mice were removed and placed on ice. One gram of tissue was homogenized in cold 0.1 M Tris buffer at pH 7.4 (1 : 10 w/v) in a tissue homogenizer (Sakura, Japan). The homogenates were centrifuged at 12000 rpm for 5 min at 4°C. Then, the extract was incubated at 37°C for 1 h with 1 mL of the homogenate solution. Afterward, 8.1% sodium dodecyl sulfate (SDS), 1.33 M acetic acid (pH 3.4), and 0.6% thiobarbituric acid (TBA) were added to the medium. The reaction mixture was incubated at 97°C for 1 h and later the absorbance was measured at 532 nm. TBARS concentrations were estimated from a standard curve of malondialdehyde bis-(dimethyl acetal) and reported as nmol MDA/mg protein. Data was expressed as percentage of inhibition. It was calculated from the absorbance values of the control and experimental tubes and IC_50_ value was calculated. Experiment was repeated three times and values were represented as mean ± S.E. of the experiments. Protein concentration was also determined [20].

### 2.5. Effects of* U. fasciata* Extract on BP-Induced Cytotoxicity in Hepatic C9 Cells

Rat hepatocytes clone 9 culture (gift from Dr. M. Marina-Silva, IFC, UNAM, Mexico) was grown in DMEM supplemented with 10% newborn calf serum, 50 U penicillin/mL, and 50 *μ*g streptomycin/mL. For subcultures, 5 × 10^5^ cells at 1 : 10 dilution were plated in a 100 mm petri dish. The medium was changed every three days and the cells were harvested at ~100% confluence with 0.25% trypsin-EDTA. For treatments, C9 cells were seeded at a density of 5,000 cells/well and allowed to grow and equilibrate for 24 h.

To explore the potential toxicity of* U. fasciata*, the cells were exposed to a range of extract concentrations (10–1000 *μ*g/mL) for 24, 48, and 72 h. For experiments conducted to evaluate cytoprotection, the concentrations of the extract tested were 1, 5, 10, 100, and 500 *μ*g/mL at a BP concentration of 10 *μ*M.

In the first experimental series, the cells were pretreated with increasing concentrations of the extract for 12 h. Then, they were treated with the extract and BP for an additional period of 6 h. In the second experimental series, the cells were treated with the extract for 12 h; they were then washed and exposed to BP or to the extract plus BP for an additional 24 h or 48 h period. A third experimental series was performed in which the cells were exposed to both the extract and BP for 24 or 48 h. Finally, cell viability was evaluated by conducting the [3-4, 5-dimethylthiazol-2-yl]-2,5-diphenyl tetrazolium bromide (MTT) assay as previously described [21]. The percentage of cell viability was calculated relative to that of the nontreated cells, which were assumed to be 100% viable.

### 2.6. Effects on CYP1A and CYP2B Isoforms in Rat Liver Microsomes

#### 2.6.1. Preparation of Liver Microsomes

For the* in vitro* assays, liver microsomes were obtained from the phenobarbital and 5,6-*β*-naphthoflavone-induced S_9_ fraction [22]. For the* in vivo* assay, microsomes were prepared from the liver of animals exposed to* Ulva fasciata* extract or controls. Livers were excised, washed, and homogenized in 0.15 M KCl solution. The homogenate was centrifuged for 10 min at 9000 ×g and the supernatant was collected (S_9_ fraction). The S_9_ fraction was further centrifuged at 100,000 ×g for 60 min and the pellet was resuspended in 0.1 M phosphate buffer (pH 7.4) and 0.25 M sucrose and centrifuged again at the same conditions. The microsomal fraction was resuspended in 0.1 M phosphate buffer (pH 7.4), 1 mM EDTA, 0.1 mM dithiothreitol (DTT), and 20% v/v glycerol. Protein concentration was determined [20] and the microsomal fraction was kept at −80°C until use.

#### 2.6.2. CYP1A and CYP2B Activities

The activities of CYP1A1-related ethoxyresorufin-*O*-deethylase (EROD), CYP1A2-related methoxyresorufin-*O*-demethylase (MROD), CYP2B1-related pentoxyresorufin-*O*-dealkylase (PROD), and CYP2B2-related benzyloxy-resorufin-*O*-dealkylase (BROD) were measured as described elsewhere [23] with minor modifications. The extract was added to the incubation mixtures as an aqueous solution. The incubation mixture containing different concentrations of the extract (or water as control), rat liver microsomes (0.01–0.1 mg), substrate (1 *μ*M ER, 5 *μ*M MR, 5 nM PR, or 20 nM BR), and buffer with pH 7.6 (50 mM Tris-HCl, 25 mM MgCl_2_) was incubated for 3 min at 37°C. The reaction was started by the addition of NADPH (0.5 mM) and was monitored for 3 min, with the fluorescence signal being recorded every 15 s. The activities were calculated from a standard curve of resorufin (5–50 pmol/mL).

### 2.7. Biochemical Characterization of the Extract's CYP1A1 Inhibitory Effect

The final reaction mixture contained 1 pmol CYP1A1 Supersome, 0.5 mM NADPH, different ER concentrations, and 0, 10, 20, 30, 40, and 50 *μ*g/mL of* U. fasciata* extract. Appropriate controls without the tested extract were established. The reaction was started with the addition of NADPH. *V*_max_ and *K*_*m*_ values were obtained from the 1/*y*- and 1/*x*-intercepts of a Lineweaver-Burk plot after incubation at 37°C for 10 min. Furthermore, a kinetic analysis was performed by using the Dixon plot and replot of the Y-intercept of the Linewaver-Burk plot.

### 2.8. Antigenotoxic Effects of the Extract on the DNA Damage Induced by BP

Male BALB/C mice (18–20 g) were obtained from Biomedical Research Institute, National University of Mexico (UNAM, Mexico, DF, México). The animals were adapted to standard conditions (temperature: 20 ± 2°C, humidity: 40–60%, and 12 h light/dark cycle) for one week. They were fed with a commercial standard rat diet and water* ad libitum*. The experiment was conducted in accordance with the ethical guidelines for investigations with laboratory animals of the Institute of Biomedical Research, UNAM. The study was conducted using 4 experimental groups (5 animals per group). The animals were orally administered 10, 100, and 250 mg/kg* U. fasciata* extract for 5 days. The control group received only distilled water (vehicle). One hour after administration of the last dose of the extract, the animals received 250 mg/kg BP by the oral route. After 24 h, the animals were sacrificed and their livers were removed, weighed, washed (0.15 M KCl), and conserved at −70°C until the microsomal fraction was obtained.

#### 2.8.1. Liver CYP1A1/1A2 Activities in Mouse Liver Microsomes

The formation of resorufin after* O*-dealkylation of 7-ethoxyresorufin and 7-methoxyresorufin were measured in microsomes from treated and control animals is as described above.

#### 2.8.2. Determination of Micronuclei (MN) Induction

Once the animals were sacrificed, both femurs were removed and freed from skin and muscle by traction. The proximal end of the femur was carefully shortened until the marrow canal became visible. One milliliter of serum was introduced into the bone canal and the femur was submerged in a centrifuge tube filled with fetal calf serum. The marrow was aspirated and flushed several times. Cells were centrifuged at 1000 rev/min for 5 min. Two drops of the cell fraction were placed onto clean, dry slides and smeared, fixed in methanol, and stained with Giemsa 5% (v/v) for 12 min. The percentage of micronucleated cells was determined using a sample of 2000 polychromatic erythrocytes (PCE). Normochromatic erythrocytes (NCE) were also scored in 2000 erythrocyte samples to determine the PCE/NCE ratio [24].

### 2.9. Statistical Analysis

Results were expressed as the mean ± SD from three independent experiments. The statistical analysis was performed by one-way ANOVA, followed by a Dunnett's test for multiple comparisons using the GraphPad Prism 5 statistical software package. Statistical significance (*p*) was set at 0.05.

## 3. Results

### 3.1. Chemical Characterization of* U. fasciata* Extract

**Figure 1:**
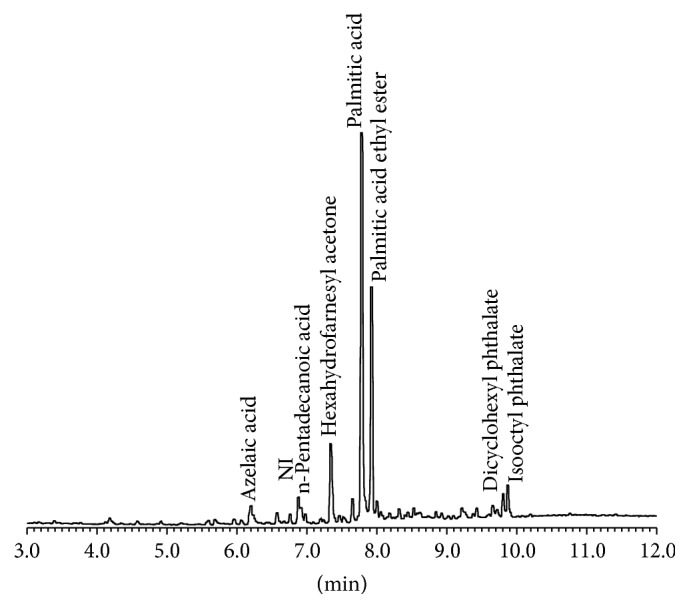
Chromatogram obtained by GC/MS analysis of the extract of* Ulva fasciata*.

The chromatogram obtained from the GC-MS analysis of the fraction obtained after elution with CHCl_3_/H_2_O (1 : 1 v/v) from the* U. fasciata* extract showed 8 chromatographic peaks from 6 to 10 min and 5 new components were identified (Figure 1, Table 1). Most of the compounds and their derivatives were acidic in nature. The main component was palmitic acid (51.3%) and its ethyl ester (24.7%). These results increase the knowledge on the phytochemical composition of* U. fasciata* extract and it should add to the understanding of the product pharmacological properties.

### 3.2. Antioxidant Capacity of* U. fasciata* Extract

**Figure 2:**
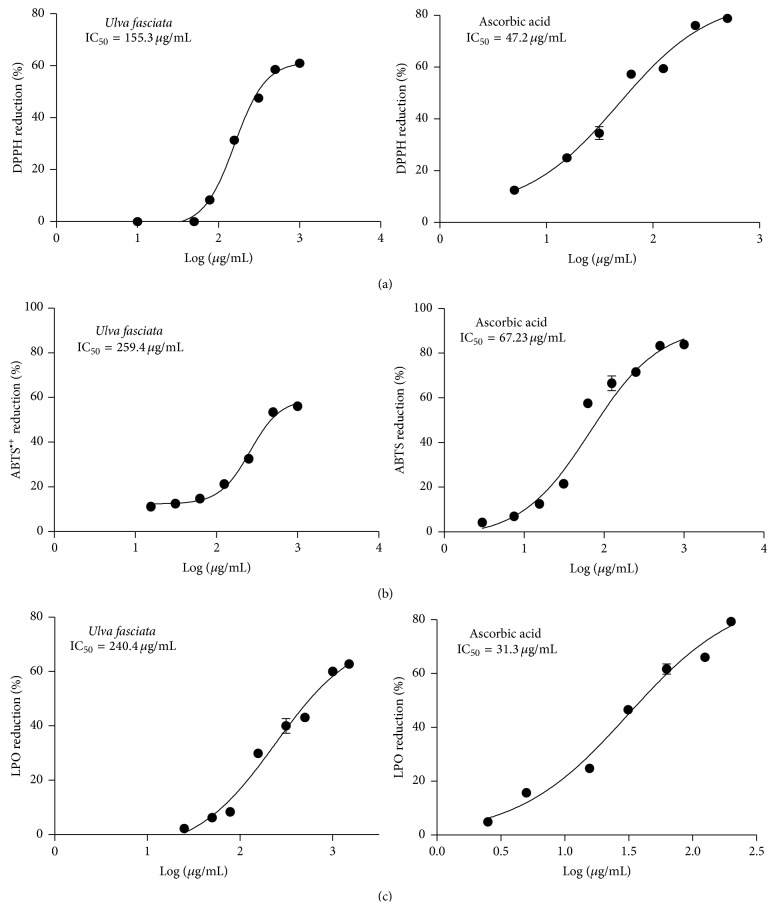
Antioxidant effects of* Ulva fasciata* extract. (a) DPPH radical, (b) ABTS^•+^ radical, and (c) lipid peroxidation assay. Results represent the mean value ± SD of three experiments carried out in triplicate. Antioxidant effectiveness was expressed as IC_50_. Ascorbic acid was used as standard. Coefficients of covariance were minor of 15 percent in all the cases.

As the antioxidant activity is usually system-dependent, three methods were used to test the antioxidant effectiveness of this product, including two based on the evaluation of the free-radical scavenging capacity of the extract in a cell-free system and one based on its effects on lipid peroxidation in liver homogenates. Results concerning the antioxidant capacity of the extract are shown in Figure 2. As it can be seen in cell-free systems, the extract showed a moderate scavenging effect, showing IC_50_ values of 155.3 *μ*g/mL and a maximum effect of 58.26% at the concentration of 500 *μ*g/mL in the DPPH assay. Meanwhile, for the ABTS^•+^ radical the IC_50_ value was in the order of 240.4 *μ*g/mL and the maximum effect was observed at 500 *μ*g/mL. In accordance with these results,* U. fasciata* inhibited lipid peroxidation when added to liver homogenates, showing IC_50_ values of 259.4 *μ*g/mL. In all the cases, the positive control (ascorbic acid) showed the expected effect (Figure 2).

### 3.3. Effects of* U. fasciata* Extract on the BP-Induced Cytotoxicity in Hepatic C9 Cells

**Figure 3:**
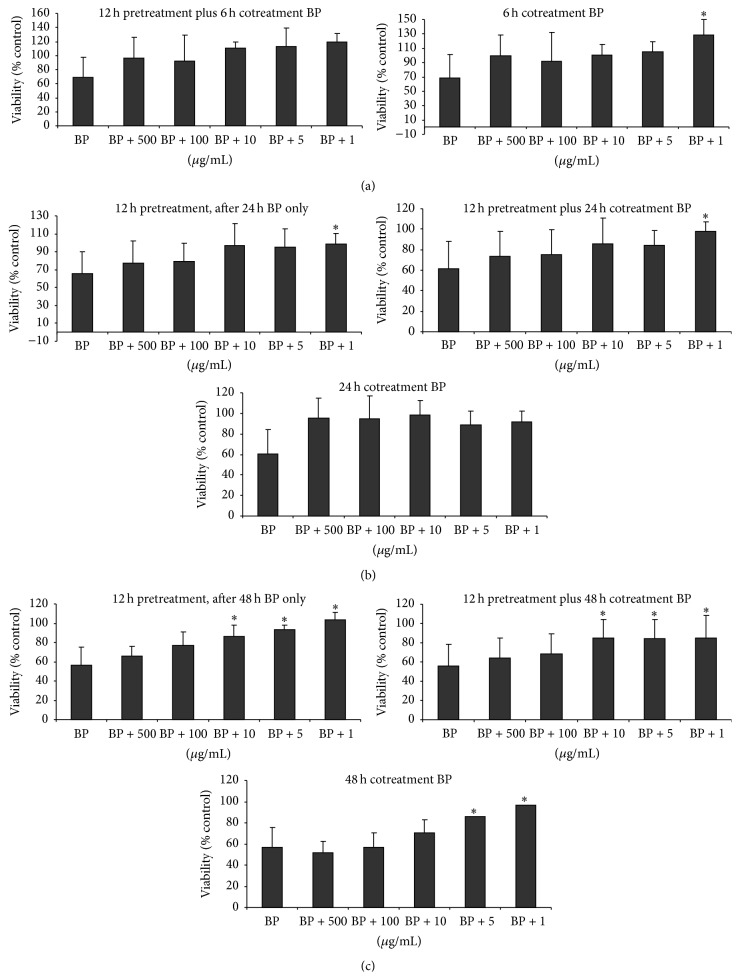
Effects of the* Ulva fasciata* extract on benzo[a]pyrene- (BP-) induced toxicity to rat C9 cells. (a) Cells were exposed for 12 h to increasing concentrations of* Ulva fasciata* extract and for an additional 6 h period in the presence of the extract and BP, or they were exposed for a 6 h period to both products. (b) Cells were exposed for 12 h to increasing concentrations of the extract; then, they were washed and exposed to the extract plus BP or BP only for an additional 24 h period. In the other condition, cells were exposed to both products for 24 h. (c) Cells were treated in conditions similar to those described in (b); however, in this case, the additional period was 48 h long. In all series, the BP concentration used was 10 *μ*M. Finally, cell viability was determined by the MTT assay. Results are expressed as the percentage of control (untreated) cells. Each point represents the mean ± SD of three experiments with three replicates. ^*∗*^ *p* < 0.05 in relation to cells treated to the toxin (Dunnett's test).

*In vitro* toxicity effects of* Ulva fasciata* extract were evaluated in the C9 cell line. No significant cytotoxic effects were observed after exposing cells to* Ulva fasciata* for 24–72 h at any of the concentrations tested (10–1000 *μ*g/mL) (data not shown). In order to evaluate the potential cytoprotection of* Ulva fasciata* against the toxicity induced by the BP, the viability of C9 cells was scored after different treatment protocols. Exposure of C9 cells to BP induced the expected reduction in cell viability; however, a noticeable recovery was observed when the cells were preexposed to the extract (Figure 3). A similar level of recovery was noted in the cells coexposed to the extract plus BP, whereas the protective effect was more evident at the lower concentrations tested (1 and 5 *μ*g/mL), being significantly different than control cells. In addition, the exposure time where the effects of the extract were more consistent was 48 h (Figure 3).

### 3.4. *In Vitro* Effect of* U. fasciata* Extract on Hepatic P450 Activities

**Figure 4:**
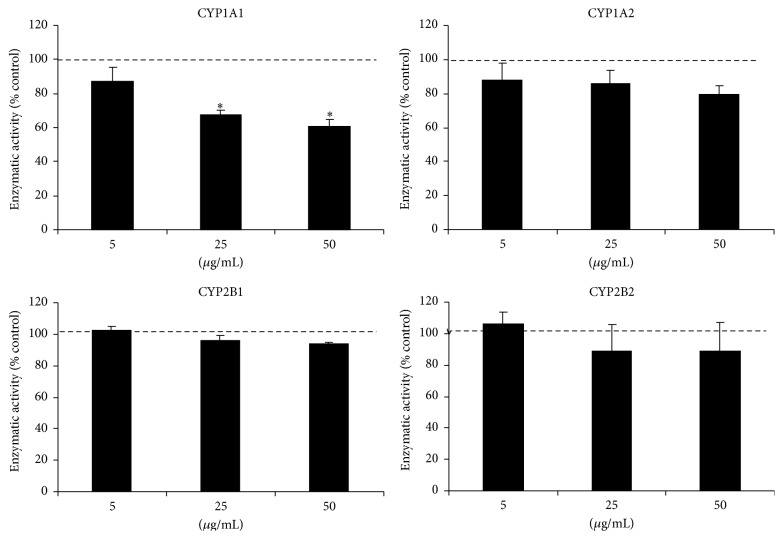
Effects of* Ulva fasciata* extract on microsomal P450 activities in rat PB-induced microsomes. P450 isoform activities were assayed in rat PB-induced microsomes incubated with appropriated substrates and* Ulva fasciata *concentrations. The values represent mean ± SEM of three independent experiments, ^*∗*^ *p* < 0.05 (Dunnett's test).

**Figure 5:**
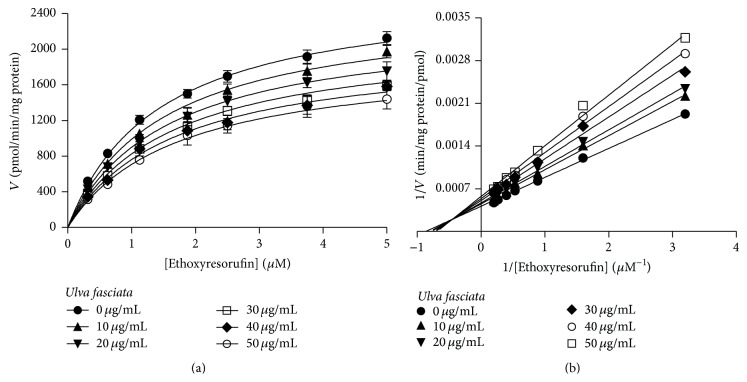
CYP1A1 associated ethoxyresorufin-*O*-deethylase activity in the absence and presence of different concentrations of* Ulva fasciata*. (a) Reaction in a final volume of 200 *μ*L was monitored for 10 min recording the fluorescence signal each 15 s. The reactions consisted of 1 pmol Supersome protein, 50 mM NADPH, and ethoxyresorufin at different concentrations. For the inhibition experiments, the extract was added at different concentrations to the reaction mixture. (b) Lineweaver-Burk plot analysis was performed to obtain the kinetic parameters. Each point in (a) represents the mean ± SD obtained from three independent experiments.

**Figure 6:**
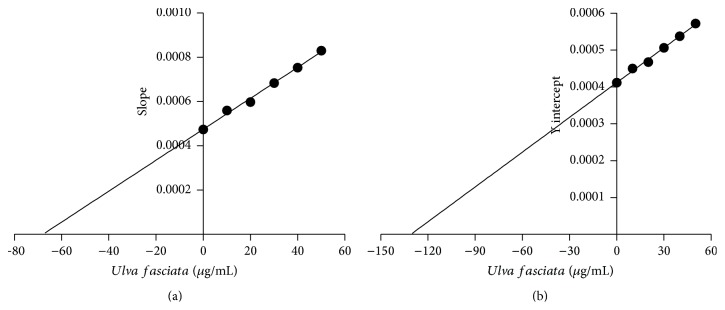
Confirmation of the CYP1A1 inhibitory properties of* Ulva fasciata* extract by (a) Dixon plot and (b) Y-intercept of the Linewaver-Burk plot versus inhibitor's concentration. Each plot was obtained from independent reactions containing the desired concentration of ethoxyresorufin (0.31–5.00 *μ*M), 1 pM Supersome protein, 50 mM NADPH, and different concentrations of the extract in a final volume of 200 *μ*L. Each reaction was followed for 10 min, with the fluorescence signal being recorded every 15 s. Each point in (a) represents the mean ± SD obtained from three independent experiments. Slope of each plot in (a) was obtained and plotted versus the inverse of the concentration of ethoxyresorufin (b).

The activities of CYP1A1, CYP1A2, CYP2B1, and CYP2B2 were measured in induced rat liver microsomes alone, or in the presence of the extract at concentrations of 5, 25, and 50 *μ*g/mL. As seen in Figure 4, the extract produced a significant reduction of 35–40% in CYP1A1-associated EROD activity with respect to the activity recorded in the controls. A slight nonsignificant inhibition was observed for CYP1A2-associated MROD activity. No appreciable changes in CYP2B activity were found. Taking into account the above-mentioned results, we decided to investigate the type of inhibition produced by the extract on CYP1A1 catalytic function. The kinetic parameters of rat recombinant CYP1A1 (Supersomes), using ethoxyresorufin as the substrate, were *V*_max_ = 2677 ± 51 pmol/min/mg protein and *K*_*m*_ = 1.44 ± 0.07 *μ*M (Table 2). CYP1A1 activity was inhibited in the presence of the extract at concentrations of 10–50 *μ*g/mL (Figure 5), with a Ki of 67.9 ± 9.3 *μ*g/mL (Table 2). Results in Figure 6 show the mixed-type inhibitory mechanism of the* U. fasciata* extract.

### 3.5. Effects of* U. fasciata* Extract on BP-Induced Genotoxicity in Male BALB/C Mice

The results in Table 3 shows that pretreatment of animals with different doses of the* U. fasciata *extract prevented an increase in the frequency of micronucleated bone marrow polychromatic erythrocytes (MNPCEs) observed in bone marrow cells of BP-exposed animals. The reductions ranged from 47 to 65%. Compared to the controls, the treated animals groups showed no significant changes in the cytotoxicity index. Additionally, no significant differences in body weight and water consumption were observed between the groups (data not shown).

**Figure 7:**
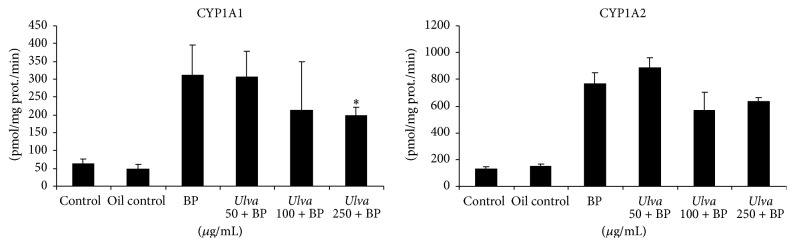
Effects of the pretreatment of* Ulva fasciata* extract on CYP1A activities in liver microsomes from male BALB/C mice exposed to benzo(a)pyrene (BP). CYP1A1 and CYP1A2 activities were assayed in liver microsomes from mice pretreated with different doses of* Ulva fasciata* or controls during 5 days and after administration of an oral dose of BP (250 mg/kg). The values represent mean ± SD; ^*∗*^ *p* < 0.05 (Dunnett's test).

On the other hand, the effects of the* U. fasciata* extract on the enzymatic activity of CYP1A1 and CYP1A2 in liver microsomes from the treated animals and controls are shown in Figure 7. Mice orally exposed to the extract for 5 days before BP exposure showed a decrease in hepatic CYP1A1 activity compared to the activity recorded in the BP-positive control group.

## 4. Discussion

The use of chemoprotective agents in everyday life has been suggested to be effective in preventing the increase of cancer frequency in human populations. For instance, many dietary antioxidants were shown to be potentially beneficial agents by reducing oxidative stress involved in the development of different chronic diseases, including cancer [25].

Natural products contain bioactive constituents that potentially block or reverse the carcinogenesis process at early stages. Therefore, modification in lifestyle habits, including diet, may lead to a reduction in the incidence of these diseases [25]. The central point of this strategy is that dietary constituents may inhibit carcinogenesis through different mechanisms. Blocking biotransformation of procarcinogens through inhibition of the CYP system is one of them. During phase I of xenobiotic metabolism, polar metabolites are formed which are substrates for phase II enzymes in order to render easily excreted products. Nevertheless, metabolites resulting from phase I may be highly electrophilic and carcinogenic, capable of interacting with DNA, and causing mutations [26, 27].

In the present study, we demonstrated the* in vitro* scavenging properties and protective effects of* U. fasciata* extract on BP-induced damage, a recognized human carcinogen. The extract itself was not cytotoxic to hepatic C9 cells but it showed a cytoprotective effect against BP-induced cytotoxicity (Figure 3). Surprisingly, lower concentrations of the extract (1 *μ*g/mL) completely abolished the cytotoxic effect of BP. Since BP exposure should give rise to electrophilic metabolites and the extract showed moderate scavenging activity measured as DPPH, ABTS, and lipid peroxidation assays (Figure 2), the cytoprotection observed may be due to a decrease of the oxidative stress in C9 cells induced by* U. faciata* extract. On the other hand, the significant* in vitro* and* in vivo* inhibitory effects of the extract on CYP1A1 activity (Figures 3 and 7) may play an important role in the protection from BP-induced damage. The extract showed mixed-type inhibition kinetics with increasing *K*_*m*_ and decreasing *V*_max_ as the extract concentration increased (Figures 5 and 6, Table 2). These results suggest that the active compound(s) in the extract might bind to both the active site of the enzyme and an allosteric site. To our knowledge, this is the first report concerning the potential chemopreventive effects of this green seaweed on DNA-induced damage.

It is known that BP induces genetic lesions such as DNA single-strand breaks, DNA–protein cross-links, and chromosomal aberrations [28]. In an attempt to demonstrate the* in vivo* antigenotoxic potential of the extract, we explored its capacity to reduce the frequency of micronuclei induced by BP. The levels of BP-induced micronuclei were found to be significantly lower in the extract pretreated animals than in those exposed only to BP (Table 3), suggesting that* U. fasciata* protects against DNA damage resulted from endogenously reactive species produced during the intermediary metabolism of BP.

Interestingly, a dose-related decrease in hepatic CYP1A activity was detected in the three extract treated groups compared to the BP-treated group (Figure 7). This finding confirmed the results of the* in vitro* inhibition study described above (Figure 3), and it reinforces the hypothesis that inhibition of metabolizing enzymes may play an important role in* U. fasciata* antigenotoxic properties.

Thus, our results indicate that prevention of BP-induced genotoxic damage by the tested extract may involve the modulation of different molecular targets, where the interaction with Phase I enzymes associated with carcinogen activation (CYP1A subfamily) could play an important role. However, the radical scavenging properties of the extract and the modulation of other antitumor mediators at the cellular level could not be discarded. In addition, our present results as well as others [16] concerning the phytochemical characterization of this extract (Figure 1) indicated that* U. fasciata* is a rich source of many micro- and macronutrients, some of which are associated with the modulation of different biomarkers involved in cancer progression.

Fatty acids and carotenoid pigments are present at high concentrations in seaweeds, including the specie of* U. fasciata* studied here. Carbohydrates, proteins, saponins, alkaloids, and flavonoids are also found to be present [29, 30]. Here, we broaden the phytochemical study of this extract by using GC-MS. The analysis performed in this study (Figure 1) showed palmitic acid to be one of the main components in the fraction analyzed, and the biological importance of this molecule has been highlighted [31]. Ryu et al. [14] reported the presence of carotenoids in the extract. Nadathur et al. [32] have reported the antimutagenic properties of palmitic acid against the direct mutagen methylnitronitrosoguanidine (MNNG). Its mechanism of action has been suggested to involve trapping of the mutagen inside the micelles formed by the fatty acid. Furthermore, the same group reported that isopalmitic acid also interferes with the mutagenicity of 7,12-dimethylbenz[a]anthracene (DMBA) by inhibiting the activity of CYP1A1. Whether or not the same mechanism of action could be considered for the antigenotoxic effect of* U. fasciata* extract reported here needs to be investigated further.

## 5. Conclusion

In summary, this study demonstrated that* U. fasciata* has protective effects against* in vitro* and* in vivo* damage induced by BP, and different pathways could be modulated by the extract. Meanwhile, its capacity for inhibiting CYP1A function seems to be the main mechanism involved. Thus, this marine green alga might be an abundant source of potential complementary and alternative functional food for the prevention of cancer and other degenerative diseases associated with xenobiotics bioactivation in the organism. The mechanisms underlying the antigenotoxic effects of* U. fasciata* deserve further investigation.

## Figures and Tables

**Table 1:** Compounds isolated from *Ulva fasciata* extract.

Compounds	RT (min)	Area (%)
Azelaic acid	6.205	3.08
NI	6.886	2.79
n-Pentadecanoic acid	6.922	1.21
Hexahydrofarnesyl acetone	7.344	11.84
Palmitic acid	7.787	51.31
Palmitic acid ethyl ester	7.928	24.67

RT: retention time %; relative percentage of the each extract constituent expressed as percentage with respect to peak area normalization.

**Table 2:** Effect of *Ulva fasciata* extract on kinetics parameters of CYP1A1 Supersome.

Kinetic parameters	*Ulva fasciata*
*V*_max_ (pmol/min/mg protein)	2677 ± 51
*K*_*m*_ (*μ*M)	1.44 ± 0.07
Type of inhibition	Mixed type
Ki	67.9 ± 9.3 *µ*g/mL
*α*Ki	130.8 ± 15.5 *µ*g/mL

**Table 3:** Effects of *Ulva fasciata* extract on micronucleus assay in Balb/C male mice.

Group (mg/kg)	IC	MN/PCE
Negative control	10.8 ± 0.28	4.3 ± 0.8
Vehicle control	2.1 ± 0.47	6.6 ± 2.6
Benzo(a)pyrene only	2.23 ± 0.75	15.3 ± 2.6
Benzo(a)pyrene + *Ulva* (50 mg/kg)	2.48 ± 0.50	7.9 ± 1.9^*∗∗*^
Benzo(a)pyrene + *Ulva* (100 mg/kg)	1.88 ± 0.25	5.3 ± 1.4^*∗∗*^
Benzo(a)pyrene + *Ulva* (250 mg/kg)	2.63 ± 0.47	5.15 ± 0.65^*∗∗*^

MN: micronucleus; PCE: polychromatic erythrocytes; 2000 cells/animal were examined; IC: cytotoxicity index: polychromatic erythrocytes/normochromatic erythrocytes; ^*∗∗*^ *p* < 0.01; comparisons between treated groups with the extract plus PB and the control group treated with BP only (Dunnett's test).
